# Evaluation of Radiographic Contrast-Induced Nephropathy by Functional Diffusion Weighted Imaging

**DOI:** 10.3390/jcm10194573

**Published:** 2021-10-01

**Authors:** Thomas Andreas Thiel, Julian Schweitzer, Taogetu Xia, Eric Bechler, Birte Valentin, Andrea Steuwe, Friedrich Boege, Ralf Westenfeld, Hans-Jörg Wittsack, Alexandra Ljimani

**Affiliations:** 1 Department of Diagnostic and Interventional Radiology, Medical Faculty, Heinrich Heine University Dusseldorf, D-40225 Düsseldorf, Germany; ThomasAndreas.Thiel@med.uni-duesseldorf.de (T.A.T.); Eric.Bechler@med.uni-duesseldorf.de (E.B.); Birte.Valentin@med.uni-duesseldorf.de (B.V.); Andrea.Steuwe@med.uni-duesseldorf.de (A.S.); Hans-Joerg.Wittsack@med.uni-duesseldorf.de (H.-J.W.); 2 Division of Cardiology, Pulmonology, and Vascular Medicine, Medical Faculty, Heinrich Heine University Düsseldorf, D-40225 Düsseldorf, Germany; Julian.Schweitzer@med.uni-duesseldorf.de (J.S.); Ralf.Westenfeld@med.uni-duesseldorf.de (R.W.); 3 Institute of Clinical Chemistry and Laboratory Diagnostics, Medical Faculty, Heinrich Heine University Düsseldorf, D-40225 Düsseldorf, Germany; Taogetu.Xia@hhu.de (T.X.); Boege@med.uni-duesseldorf.de (F.B.)

**Keywords:** functional renal imaging, DWI, DTI, radiographic contrast medium, kidney, CIN

## Abstract

Contrast-induced nephropathy (CIN) resembles an important complication of radiographic contrast medium (XCM) displayed by a rise in creatinine levels 48–72 h after XCM administration. The purpose of the current study was to evaluate microstructural renal changes due to CIN in high-risk patients by diffusion weighted (DWI) and diffusion tensor imaging (DTI). Fifteen patients (five CIN and ten non-CIN) scheduled for cardiological intervention were included in the study. All patients were investigated pre- and post-intervention on a clinical 3T scanner. After anatomical imaging, renal DWI was performed by a paracoronal echo-planar-imaging sequence. Renal clinical routine serum parameters and advanced urinary injury markers were determined to monitor renal function. We observed a drop in cortical and medullar apparent diffusion coefficient (ADC) and fractional anisotropy (FA) before and after XCM administration in the CIN group. In contrast, the non-CIN group differed only in medullary ADC. The decrease of ADC and FA was apparent even before serum parameters of the kidney changed. In conclusion, DWI/DTI may be a useful tool for monitoring high-risk CIN patients as part of multi-modality based clinical protocol. Further studies, including advanced analysis of the diffusion signal, may improve the identification of patients at risk for CIN.

## 1. Introduction

The application of iodine-containing radiographic contrast medium (XCM) is mandatory in many modern diagnostic and therapeutic procedures, including computed tomography (CT) scans, interventional radiology, vascular surgery, and interventional cardiology. XCM is eliminated through the kidneys, leading to a complication referred to as contrast-induced nephropathy (CIN). The Kidney-Disease-Improving-Global-Outcomes (KDIGO) and the Acute-Kidney-Injury-Network (AKIN) guidelines [1] classify CIN criteria as non-oliguria (<0.5 mL/kg/h for 6–12 h) and a rise in serum creatinine (>0.3 mg/dL or 1.5–2 of the base-line value) 48–72 h following XCM administration. Depending on the definition and the characteristics of the population studied, the reported incidence of CIN ranges from 10% to 30% [2,3,4]. In recent decades, individual patient risk factors for CIN prior to intervention were discussed and published in the form of various scoring systems [2,5,6,7,8,9]. These scoring systems include parameters such as age, gender, comorbidities such as heart failure, diabetes mellitus, pre-existing renal insufficiency indexed by serum creatinine, as well as the type of medical procedure planned. However, this practice is associated with inappropriately; ow rates of potential life-saving interventions in the patient cohort at risk for CIN due to an unfortunate trend coined “renalism” [10].

Evidently, CIN deteriorates patient prognosis, causes longer hospitalization, and ultimately leads to higher costs [11]. Physiologically, XCM induces vasocontraction and a rise of blood osmolarity in renal arteries. This leads to perfusion reduction, hypoxia, and ischemia, especially in the proximal and distal tubular system of the medulla [3,12]. Furthermore, the increased absorption of XCM into the tubular interstitial space is toxic for the tubular cells and leads to the apoptosis and necrosis of the tubular cells [13]. Currently, the only CIN prophylaxis consists of adequate hydration of patients with eGFR < 30 mL/min prior to the application of XCM [14]. However, this can be very challenging in patients with combined cardiac and renal insufficiency due to the need for volume monitoring and is currently controversial, as discussed in the literature [15,16]. Furthermore, individual kidney protective approaches for the CIN-management are also discussed widely [14,15,17,18,19]. For these individual approaches, a further stratification of high-risk CIN-risk patients after application of XCM is important. In this regard, advanced urinary injury markers, e.g., neutrophil gelatinase-associated lipocalin (NGAL), Dickkopf-3 (DKK3), tissue inhibitor of metalloproteinase 2 (TIMP2), insulin-like growth factor binding protein 7 (IGFBP-7), and kidney injury molecule-1 (KIM-1), which are considered to reflect tubular and glomerular renal damage more precisely, were recently introduced for monitoring acute kidney injury (AKI) [20,21,22,23,24]. However, the urinary injury markers are based on ELISA (Enzyme-linked Immunosorbent Assay) analysis. ELISA leads to higher costs for the urinary analysis and, importantly, excludes ad-hoc kidney monitoring, making advanced urinary injury markers unsuitable for quick diagnosis.

In conclusion, one of the main issues in management of CIN is still unsolved: the delayed diagnosis of CIN after 48–72 h, during which time renal damage has already progressed. Diagnostic tools identifying high-risk CIN patients with still reversible renal damage might improve the outcome of these patients and reduce the aforementioned clinical “renalism”. Non-invasive, functional renal MRI biomarkers might be a promising approach in this regard [25]. In particular, diffusion weighted imaging (DWI) and diffusion tensor imaging (DTI) have been shown to potentially reflect acute changes in renal microstructure in animal and human studies [26,27].

The purpose of the current study is to evaluate microstructural renal changes by diffusion weighted imaging (DWI) and diffusion tensor imaging (DTI) triggered by CIN in high-risk patients undergoing cardiological interventional treatment with XCM administration.

## 2. Materials and Methods

### 2.1. Patient Collective

The local ethics committee approved the study, and written informed consent was obtained from all patients. A total of 15 patients (5 females and 10 males, mean age 76.3 ± 8.1 years, range 54 to 84 years) with known history of chronic kidney disease (CKD) and Mehran score > 10 [5] were included in the study. All patients planned to undergo cardiological interventional treatment with the administration of iodine-containing XCM. Following the recommendations for XCM application in high risk patients [19,28,29], all patients received an iodine-containing, low-osmolality (0.64 Osm/kg H2O) XCM (Accupaque 300 mg J/mL, GE Healthcare Buchler GmbH & Co.KG, Germany) [30]. Renal protective hydration before the intervention was performed in all patients according to the current guidelines [14,15,18]. 

All patients were investigated twice, prior to the intervention to obtain renal MRI baseline parameters and shortly after intervention to obtain possible renal microstructural changes due to the XCM administration.

### 2.2. MRI Protocol

Both MRI measurements were performed with the same clinical 3T MR-scanner (MAGNETOM Skyra Siemens Healthcare, Erlangen, Germany) in the supine position using an 18-channel body and a 30-channel spine coil integrated in the patient table.

For anatomical imaging, four T2-weighted HASTE (Half-Fourier Acquired Single-Shot Turbo Spin Echo) sequences were acquired with following imaging parameters: 

Coronal: 40 slices; slice thickness: 5 mm; field of view (FOV): 380 × 380 mm^2^; TR/TE: 1200/99 ms; matrix: 512 × 358; parallel imaging factor: 2.

Axial: 40 slices; slice thickness: 4 mm; field of view (FOV): 380 × 297 mm^2^; TR/TE: 1400/96 ms; matrix: 320 × 259; parallel imaging factor: 2.

Sagittal: 2 × 5 slices; slice thickness: 4 mm; field of view (FOV): 380 × 297 mm^2^; TR/TE: 1400/96 ms; matrix: 320 × 259; parallel imaging factor: 2.

Paracoronal: 40 slices; slice thickness: 5 mm; field of view (FOV): 380 × 380 mm^2^; TR/TE: 1200/99 ms; matrix: 512 × 358; parallel imaging factor: 2.

For diffusion weighted imaging, a paracoronal EPI (Echo Planar Imaging) sequence was acquired with following parameters: 15 slices; slice thickness: 5 mm; FOV 400 × 400 mm^2^; TR/TE 3000/78 ms; matrix size 176 × 176; b—values 0, 50, 400, 800 s/mm^2^; 6 diffusion weighted directions.

No respiratory or cardiac triggering was used for the acquisition. The total acquisition time was 7.28 min.

### 2.3. Post-Processing

Apparent diffusion coefficient (ADC) and fractional anisotropy (FA) parameter maps were calculated inline. Regions of interest (ROI)-based analyses of the parameter maps were performed for all datasets by two experienced radiologists, who were blinded to the patients’ clinical conditions, immediately following the MRI examination, (B.V. 4 years; A.L. 11 years of experience in renal imaging, respectively). A continuous ROI including the entire cortex (140 ± 11 pixel) as well as 5 medullar ROIs (16 ± 4 pixel each) were drawn in a central slice of each kidney, respectively.

### 2.4. Clinical Parameters

In addition to the MRI scans, a total of five blood and urine samples were collected from all patients for the clinical monitoring of renal function: one day prior (sample 1), on the day of the intervention (sample 2), and on three following days after the intervention (sample 3, 4, 5). Additionally, routine serum parameters eGFR, creatinine and cystatin C, along with advanced urinary injury markers, NGAL, DKK-3, KIM-1, TIMP-2, and IGFBP7 were determined in urine samples.

### 2.5. Statistical Analysis

Statistical analyses of all acquired MRI and clinical parameters were carried out in R (V 4.0.4, R Core Team 2021). The normal distribution of all data was tested using the Kolmogorov–Smirnov test. Subsequently, all pre- and post-interventional data were analyzed using Student *t*-tests.

## 3. Results

### 3.1. Clinical Characteristics

All image acquisitions pre and post the intervention were completed successfully in all 15 patients. MRI measurements were performed 4.6 ± 2.2 h pre and 5.1 ± 2.4 h post intervention, respectively. The clinical characteristics of the patients are summarized in Table 1.

Five out of 15 (33%) patients had alterations of serum creatinine > 0.3 mg/dL (AKIN stage I) after the administration of XCM (Table 1). One of five patients had severe kidney damage with a transient loss of kidney function and required dialysis (AKIN stage III) (Table 1). Considering the renal damage after the intervention, the patients were divided into two groups: patients without renal involvement (non-CIN, *n* = 10) and CIN group (n = 5).

The amount of XCM applied per kg of body weight did not differ significantly between non-CIN and CIN patients, at 1.8 ± 0.7 mL/kg and 1.9 ± 0.5 mL/kg, respectively (Table 1).

According to the definition of CIN, a significant difference was determined between the pre- and post-interventional clinical routine serum parameters, eGFR, creatinine, and cystatin C in the CIN group (*p* < 0.05). The pre- and post-interventional clinical renal routine parameters of the non-CIN group were not significantly different (*p* > 0.05) (Table 1). Changes of renal microstructure due to the application of XCM were also reflected by the advanced urinary injury markers for tubular damage (NGAL, DKK-3 and KIM-1) and glomeruli damage (TIMP-2 and IGFBP-7). The available advanced urinary injury markers in patients were significantly different pre- and post-XCM, respectively (*p* < 0.05). However, a statistical differentiation between non-CIN and CIN group for the advanced urinary injury markers was not possible due to the limited availability of the parameters in the CIN patients (Table 1).

### 3.2. MRI Parameters

Table 2 summarizes the acquired cortical and medullar diffusion values for non-CIN and CIN patients pre- and post-XCM administration. The mean cortical and medullar diffusion values for CIN and non-CIN patients were not significantly different (*p* > 0.05). However, in the CIN group, a significant drop of cortical and medullar ADC and FA values pre- and post-XCM administration was identified (*p* < 0.05). In contrast, the non-CIN group showed significant differentiation pre- and post-XCM for the medullary ADC values (*p* < 0.05). All other diffusion values were not significantly different pre- and post-intervention (*p* > 0.05).

**Figure 1:**
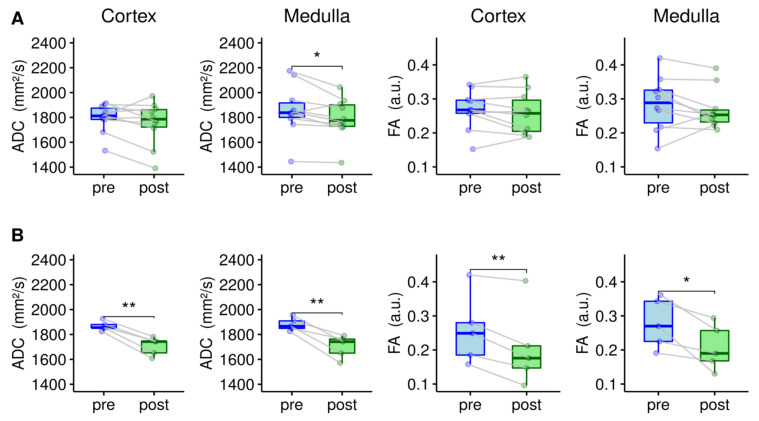
Boxplots of cortical and medullar apparent diffusion coefficient (ADC) and fractional anisotropy (FA) values pre- and post-XCM. Patients were divided into two groups (non-Contrast-Induced Nephropathy (non-CIN), *n* = 10 (**A**); CIN, *n* = 5 (**B**)). Points indicate the values for each individual and boxplots represent the mean over all patients of the group. Lines connecting the individual points represent the pre/post ADC and FA difference of the single patients. For the non-CIN group, only pre- and post-medullar ADC values were significantly different (* *p* < 0.05). All other diffusion parameters of the non-CIN patients did not differ significantly pre- and post-XCM administration. A significant drop of all diffusion parameters was identified for the CIN group (* *p* < 0.05). ** *p* < 0.01.

Boxplots displaying the mean cortical and medullar diffusion values, ADC and FA for non-CIN (A) and CIN (B) patients, respectively, are shown in Figure 1.

**Figure 2:**
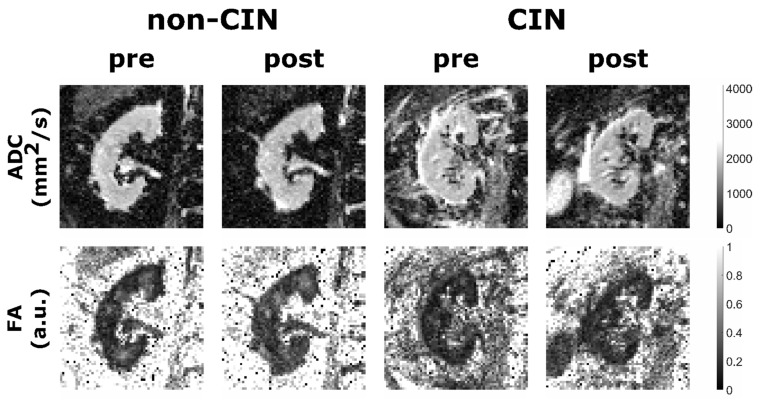
Examples of apparent diffusion coefficient (ADC) (first row) and fractional anisotropy (FA) (second row) parameter maps of a non-Contrast-Induced Nephropathy (non-CIN) and a CIN patient pre- and post-XCM. First two columns (patient 14): 82-years old male non-CIN patient with stable renal function measured by eGFR (62 to 69 mL/min), creatinine (1.11 to 1.02 mg/dL), and cystatin C (1.37 to 1.31 mg/L) in the post-interventional period. Second two columns (patient 13): 84-years old male CIN patient with a significant alteration of eGFR (71 to 49 mL/min), creatinine (0.97 to 1.33 mg/dL), and cystatin C (1.37 to 1.93 mg/L) 48 h post XCM administration.

Figure 2 shows an example of ADC and FA parameter maps of a non-CIN and CIN patient pre- and post-XCM, respectively.

Pearsons correlation index could not identify a significant correlation between acquired functional renal MRI parameters and the amount of the XCM applied during the intervention (*p* > 0.05).

## 4. Discussion

CIN is a common complication after XCM administration, especially in high-risk patients characterized by multiple comorbidities, older age, and pre-existing renal insufficiency. Oliguria and a rise of serum creatinine 48–72 h post XCM are typical symptoms of renal structural changes, which can lead to a temporal or permanent loss of kidney function and are strongly linked to patients’ outcome. Early (<48 h) diagnosis and identification of patients-at-risk for CIN are desirable to prevent irreversible damage of the renal microstructure. In the current study, we evaluated renal functional diffusion imaging, DWI and DTI, to identify patients at risk for CIN after XCM administration during cardiological intervention. Our study shows the potential of DWI and DTI to discriminate patients developing CIN from patients without renal complications.

A significant drop of the cortical and medullar pre- and post-interventional diffusion parameters ADC and FA were detected in patients with CIN after XCM administration in the current study (*p* < 0.05). MRI measurements were performed 5.1 ± 2.4 h after intervention. In the same patients, the clinical manifestation of CIN could be detected by a decrease of eGFR and increase in creatinine (>0.3 mg/dL) 48–72 h post intervention. A comparison of pre- and post-interventional periods reveals the potential of functional renal diffusion MRI for the early detection of CIN and may help facilitate the appropriate therapy in at- risk patients. In our study, 33% of the patients showed alterations of renal function after XCM administration (minimum AKIN stage I [1]), which is in the expected range for this patient cohort [2,3,4]. Only older and multi-morbid patients were included in the current study, who are at high risk for the development of renal complications due to a high Mehran score (>10 out of 11 for all patients in the current study) [5]. This may be the reason for the high renal complication rate of the current study. Overall, the diffusion values ADC and FA of the current study are consistent with the reported values in the literature for patients with impaired renal function [27,31].

XCM administration leads to a reduction of renal perfusion and tubular flow and is toxic to tubular cells, leading further to tubular necrosis [13,19,28]. We hypothesize that the significant drop of the medullar diffusion parameters ADC and FA in CIN patients might be due to XCM induced toxic oedema of tubular cells and could mark the beginning of tubular necrosis [11,13]. Both the significant differences of medullar ADC and the lack of serious renal complications, such as tubular necrosis in non-CIN patients, reflected by insignificant difference between pre and post medullar FA values can be explained by this hypothesis as well. Considering previous studies in mice [13,26] and the physiology of CIN development [3,12], a significant drop of cortical diffusion parameters can possibly be explained by the reduction of renal perfusion, especially in afferent and efferent arterioles. The addition of further functional renal imaging techniques to evaluate renal perfusion, e.g., arterial spin labelling (ASL) [32,33] or blood-oxygen level dependent (BOLD) [34,35], might lead to a better analysis of the underlying pathology and may improve the identification of patients at risk for CIN.

Another possible explanation for diffusion signal alterations might be the remaining hyperosmolar XCM in the tubular system. Several phantom studies demonstrated a change in MR signal intensity and especially in ADC values [36,37,38] after the application of XCM, indicating a relation between ADC decrease and the remaining XCM amount. Therefore, a significant decrease of renal MRI diffusion parameters in CIN patients might be an indicator for the prolonged passage of XCM through the tubular system in these patients, leading to tubular damage and finally different stages of AKIN. The lack of correlation between MRI diffusion parameters and the amount of XCM applied during the intervention in the high-risk patient cohort of the current study show that the measurement of prolonged XCM passage by MRI diffusion parameters might help to identify patients at risk for CIN development.

Clinical renal routine parameters, such as eGFR, creatinine, and cystatin C, are indirect renal functional parameters and reflect the renal function with a temporal delay [39,40]. Advanced urinary injury markers for tubular (NGAL, DKK-3 and KIM-1) and glomerular damage (TIMP-2 and IGFBP-7) were recently introduced for monitoring acute kidney injury [20,21,22,23,24]. These biomarkers reflect the cell damage more specifically as they are expressed directly in the tubular or glomerular cells and released following renal damage [41,42]. Changes of the renal microstructure due to the application of XCM were also reflected by advanced urinary injury markers in the current study, which were significantly different pre- and post-XCM in available patients (*p* < 0.05). However, recent multi-center studies consider advanced urinary injury markers as controversial for the prediction of patient outcomes [43,44,45]. The determination of urinary injury markers is based on complex, time consuming, and expensive ELISA analysis. The complexity of the urinary analysis is certainly a disadvantage of this method, which prevents a quick diagnosis of CIN and is the reason for the incomplete availability of urinary injury markers in some patients in the current study.

Not only the diagnosis but also the therapy of CIN remains challenging. All patients in the current study received prophylactic hydration prior to the application of XCM [14]. However, five of fifteen high-risk patients still showed alterations of renal function (AKIN stage I) and one of five patients needed dialysis (AKIN stage III), demonstrating the clear disadvantage of this unselective renal protective approach [15,16]. More individual therapeutic approaches are discussed in the literature [14,15,17,18,19]. To enable an individual therapy, an identification of patients at risk out of the high-risk collective is necessary shortly after XCM administration. Based on the results of the current study, functional renal diffusion MRI might be a possible tool in this regard, e.g., as a part of a multi-modality-based score system, including clinical information, renal routine parameters, and advanced urinary injury markers. However, there are limitations of MRI as well that need to be considered. Firstly, the availability of scan time to perform MRI scans twice a day could be a challenge. Secondly, possible MRI contraindications of multi-morbid patients could limit clinical use in this patient cohort. However, if the MRI examinations are limited to a selected patient cohort (e.g., Mehran score > 10) enough scan slots might be available. The individual treatment of patients-at-risk through this possible prevention of CIN can shorten hospitalization times and reduce overall costs.

A further limitation of the current study is the employed scan protocol; since two examinations need to be performed within short time in an older and multi-morbid patient cohort, the MRI acquisition protocol was kept as short as reasonably achievable.

Consequently, only a limited number of b-values was acquired. Acquisition of more b-values would allow a more precise analysis of the diffusion signal decay and potentially increase the level of discrimination between patients-at-risk for CIN and patients without complications. Further, no respiratory triggering was used during the acquisition time. In particular, after the intervention, patients tend to breath irregularly, due to potential discomfort and retirement. Respiratory triggering might improve the image quality, but also significantly increase the acquisition time. An optimization of the acquisition protocol considering the clinically justified examination time should be the subject of further studies.

In conclusion, the results of the current study show the potential of renal diffusion weighted imaging to identify patients at risk for CIN development in a high-risk patient cohort. Pathological changes in the DWI parameters seem to occur before alterations in laboratory parameters like eGFR or creatinine appear. Further studies are necessary to optimize the acquisition protocol for probing renal microstructure with a short acquisition time.

## Figures and Tables

**Table 1:** Characteristics of all patients. Gender (female (F), male (M)), age, amount of radiographic contrast medium (XCM) applied (in mL), patients’ weight (in kg), and Acute-Kidney-Injury-Network (AKIN) stage of contrast-induced nephropathy (CIN) due to Kidney-Disease-Improving-Global-Outcomes (KDIGO) [1,17] are shown. Furthermore, clinical renal routine parameter eGFR (mL/min), creatinine (mg/dL), and cystatin C (mg/L) and advanced urinary injury markers, NGAL, DKK-3, KIM-1, TIMP-2, IGFBP-7 (ng/mL) before and at the time point of the strongest measured deviation (48–72 h) after interventional are displayed.

Patient.	Gender	Age	XCM	Weight	AKIN Stage of		eGFR	Cystatin C	Creatinine	NGAL	DKK-3	KIM-1	TIMP-2	IGFBP7
#			(mL)	(kg)	CIN		(mL/min)	(mg/L)	(mg/dL)	(ng/mL)	(ng/mL)	(ng/mL)	(ng/mL)	(ng/mL)
1	M	77	162	69	-	Pre	64	1.66	1.11	-	-	-	-	-
						Post	50	1.99	1.35	-	-	-	-	-
2	F	77	200	86	III	Pre	26	2.22	1.84	-	-	-	-	-
						Post	0	3.18	4.57	-	-	-	-	-
3	F	80	80	50	-	Pre	68	1.18	0.82	7.911	0.930	0.215	0.531	0.125
						Post	77	1.19	0.74	23.730	1.096	0.897	1.260	0.212
4	M	83	135	82	I	Pre	41	1.84	1.55	10.002	0.000	0.063	0.016	0.026
						Post	29	1.89	2.07	55.970	0.148	0.268	0.551	0.096
5	F	79	160	62	-	Pre	51	1.31	1.08	26.253	0.040	0.236	0.300	0.191
						Post	59	1.17	0.88	164.211	0.269	1.629	0.799	0.794
6	M	71	130	143	-	Pre	50	1.85	1.41	10.699	0.523	1.102	1.035	0.200
						Post	52	1.77	1.38	50.213	1.321	3.547	1.260	0.794
7	M	78	190	82	-	Pre	53	1.57	1.28	30.174	0.005	0.000	0.027	0.000
						Post	54	1.55	1.26	150.336	4.197	0.933	1.260	0.228
8	M	60	69	115	-	Pre	39	1.76	1.82	27.594	0.202	0.308	0.489	0.051
						Post	41	1.68	1.76	53.215	0.358	1.204	0.729	0.432
9	M	76	140	80	-	Pre	82	1.26	0.91	3.757	0.036	0.000	0.000	0.000
						Post	83	1.27	0.88	35.355	0.624	0.630	0.831	0.018
10	F	77	144	70	I	Pre	63	1.60	0.89	23.612	0.000	0.000	0.025	0.000
						Post	42	2.04	1.23	184.652	1.783	0.154	1.260	0.277
11	M	54	290	100	-	Pre	27	2.50	2.57	-	0.000	-	0.000	0.511
						Post	33	2.03	2.18	-	0.535	-	0.982	0.799
12	M	81	90	79	I	Pre	30	3.46	2.00	-	0.000	-	0.557	0.639
						Post	22	4.52	2.59	-	0.879	-	1.013	0.989
13	M	84	160	72	I	Pre	71	1.37	0.97	-	0.000	-	0.492	0.991
						Post	49	1.93	1.33	-	2.471	-	1.109	2.156
14	M	81	145	96	-	Pre	62	1.37	1.11	-	-	-	-	-
						Post	69	1.31	1.02	-	-	-	-	-
15	F	80	227	58	-	Pre	42	1.22	1.16	-	-	-	-	-
						Post	46	1.49	1.14	-	-	-	-	-

**Table 2:** Mean cortical and medullar diffusion values (apparent diffusion coefficient (ADC), fractional anisotropy (FA)), and standard deviation pre- and post-administration of iodine-containing XCM are displayed. A significant drop of cortical and medullar ADC and FA values was identified for the contrast-induced nephropathy (CIN) group (*p* < 0.05).

		ADC (×10^−3^ mm^2^/s)		FA (a.u.)	
		Cortex	Medulla	Cortex	Medulla
non-CIN (*n* = 10)	pre	1795.7 ± 114.8	1859.2 ± 204.7	0.27 ± 0.06	0.29 ± 0.08
	post	1753.2 ± 176.0	1793.6 ± 165.2	0.26 ± 0.06	0.27 ± 0.06
CIN (*n* = 5)	pre	1868.2 ± 38.4	1880.6 ± 51.2	0.26 ± 0.10	0.28 ± 0.07
	post	1706.2 ± 72.4	1703.2 ± 90.7	0.21 ± 0.12	0.21 ± 0.07

## Data Availability

The data presented in this study are available on request from the corresponding author.
